# Treatment of myopic choroidal neovascularization: a network meta-analysis and review

**DOI:** 10.1007/s00417-023-06271-2

**Published:** 2023-11-11

**Authors:** Laura Glachs, Stefan Embacher, Andrea Berghold, Brigitte Wildner, Monja Michelitsch, Anna Tscherne, Andreas Wedrich, Laura Posch-Pertl

**Affiliations:** 1 Department of Ophthalmology, Medical University of Graz, Auenbruggerplatz 4, 8036 Graz, Austria; 2 Institute for Medical Informatics, Statistics and Documentation, Medical University of Graz, Auenbruggerplatz 2, 8036 Graz, Austria; 3 University Library, Medical University of Vienna, Währinger Gürtel 18–20, 1090 Vienna, Austria

**Keywords:** Aflibercept, Anti-VEGF, Bevacizumab, Choroidal neovascularization, Conbercept, Myopia, Myopic CNV, Photodynamic therapy, Ranibizumab

## Abstract

**Purpose:**

This is, to our knowledge, the first network meta-analysis aiming to compare all treatment modalities for myopic choroidal neovascularization (CNV).

**Methods:**

After the electronic databases were searched, two independent reviewers screened titles, abstracts, full-texts, and extracted information. Primary endpoints were change in visual outcome and central retinal thickness. We used a network meta-analysis to compare treatment outcomes in the early (≤ 6 months) and late (> 6 months) phase.

**Results:**

We included 34 studies (2,098 eyes) in our network meta-analysis. In the early phase, the use of anti-VEGF led to a gain of 14.1 letters (95% CI, 10.8–17.4) compared to untreated patients (*p* < 0.0001), 12.1 letters (95% CI, 8.3–15.8) to photodynamic therapy (PDT) (*p* < 0.0001), 7.5 (95% CI, 1.2–13.8) letters to intravitreal triamcinolone acetonide (TCA) (*p* = 0.019), and − 2.9 letters (95% CI, − 6.0–0.2) to the combination of anti-VEGF and PDT (*p* = 0.065). In the later phase, these results were largely maintained. There were no significant differences in visual outcomes between patients treated with 1 + PRN and 3 + PRN. However, the 1 + PRN group received 1.8 (SD 1.3), while the 3 + PRN group received 3.2 (SD 0.9) injections within 12 months (*p* < 0.0001).

**Conclusion:**

This network meta-analysis confirms that anti-VEGF is the most effective treatment for myopic CNV using the 1 + PRN treatment strategy.

**Supplementary Information:**

The online version contains supplementary material available at 10.1007/s00417-023-06271-2.


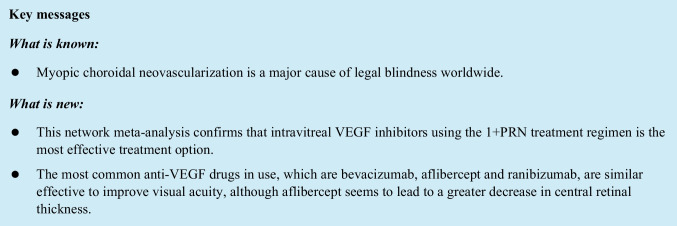


## Introduction

Pathologic myopia is a major cause of blindness affecting almost 2% of the population worldwide. Although the definition of pathologic myopia has not been standardized yet, it is usually classified as a refractive error of less than − 6.00 diopters and an axial length of ≥ 26.5 mm combined with degenerations of the sclera, choroid, and retina [1]. One of the most common complications of pathologic myopia leading to blindness is the development of choroidal neovascularization (CNV). Myopic CNV is associated with a poor prognosis if untreated leading to a decline in visual acuity. More than a third of the patients affected by myopic CNV are at risk of developing myopic CNV in the unaffected eye within 8 years [2].

For a long time, verteporfin photodynamic therapy (PDT) was the only treatment approved for myopic CNV [3]. PDT treatment was able to stabilize visual acuity; however, long-term results were discouraging. The development of VEGF inhibitors revolutionized treatment of myopic CNV and soon superseded PDT as the new gold standard treatment [4].

Several studies [5–7] have been performed to compare different treatments for myopic CNV; however, no common comparator was used. Therefore, this study is aimed at comparing the efficacy of different treatment options for myopic CNV using a network meta-analysis.

## Methods

### Literature search

**Fig. 1:**
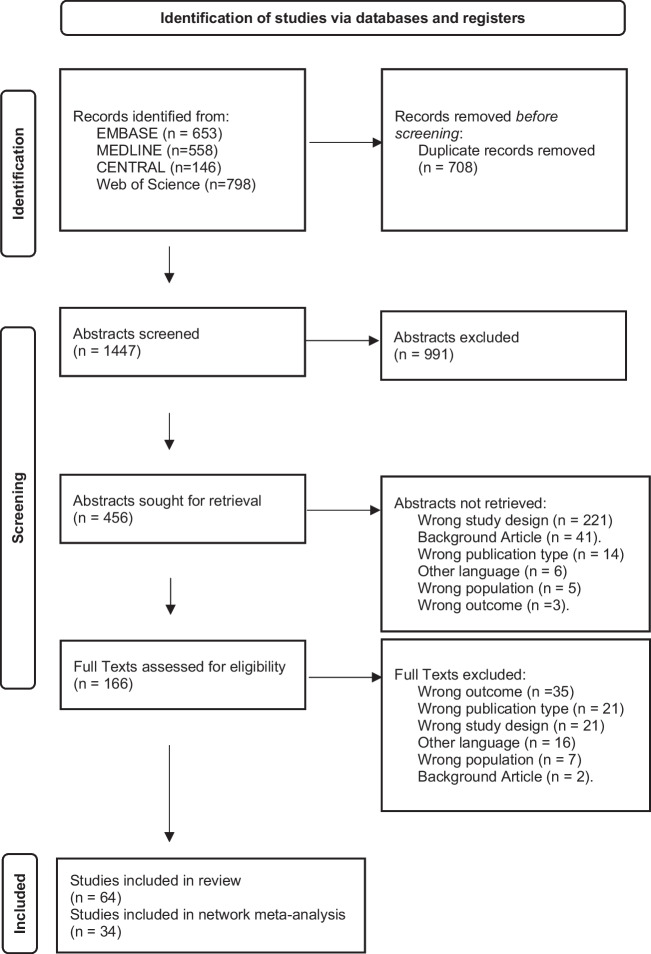
PRISMA flow diagram adapted by Page et al. [9]

The literature search was performed by an experienced medical information specialist (BW). The following electronic databases were searched for publications from database inception to July 2020: MEDLINE, Embase, Cochrane Central Register of Controlled Trials and Web of Science (SCI-Expanded, SSCI, CPCP-S and ESCI) using free term and controlled term formulations. Databases were searched for the following keywords: “myopic choroidal neovascularization” AND “treatment”;—AND “aflibercept”;—AND “bevacizumab”;—AND “ranibizumab”;—AND “conbercept”;—AND “PDT”;—AND “photodynamic therapy”;—AND “triamcinolone”;—AND “surgery”;—AND “sham”. We limited our search to articles published in English. The bibliographies of identified articles were scanned to identify additional manuscripts that were missed in our previous database search. The protocol of this network meta-analysis was not registered in PROSPERO. This review followed the Cochrane handbook [8] and the PRISMA for network meta-analysis checklist (see Fig. 1 and Supplementary Table 1) [9].

### Study eligibility criteria

All study types (i.e., randomized controlled, prospective and retrospective cohort studies, cross-sectional, case–control, and survey and surveillance reports) comparing treatments for myopic choroidal neovascularization were included. Studies had to report ≥ 2 treatment groups, original data on adult patients (≥ 18 years), and a sample size ≥ 10 and had to be published in English.

Abstracts and conference proceedings not published in peer-reviewed journals were not included.

### Study selection

Two reviewers (LP and LG) independently screened references for inclusion. Included references underwent dual abstract and subsequent full-text review to decide on final inclusion or exclusion of the study. Disagreements were resolved by discussion. The online software “Rayyan” [10, 11] was used for abstracts and full-text screening.

### Data extraction

Two investigators (LP and LG) independently extracted the title, name of authors, year of publication, study design, sample size, treatment, best-corrected visual acuity (BCVA) at baseline and follow-up, central retinal thickness (CRT) at baseline and follow-up, number of treatments, and demographic data. BCVA has been converted to ETDRS letters to enable comparison between the different ways of reporting. Further, descriptive data such as country of origin, definition of myopic CNV, minimal axial length, inclusion and exclusion criteria, and pretreatment were documented. These data were recorded in a Microsoft Excel (Microsoft Cooperation) spreadsheet.

### Data analysis

For the analysis, the change in visual outcomes was used, which is given by the mean difference between baseline and follow-up for each treatment group. Most included studies provided means and standard deviations at baseline and for specific follow-up dates. Then, the mean difference can be easily calculated, and for the standard deviation of change, the Cochrane Handbook for Systematic Reviews of Interventions [12] was followed, assuming a correlation of 0.6. This value was chosen due to a Methods Research Report [13] that refers to a median of 0.59 for correlation of change from baseline. Furthermore, one of the included studies [14] reported a correlation of 0.646. As a sensitivity analysis, no correlation was assumed. If not directly specified, further measures were taken into account to calculate the change and the corresponding standard deviation in visual outcomes. This included the use of *p* values, confidence intervals, and as a final option, if the standard deviation for the baseline was given but the standard deviation for the follow-up date was missing, the baseline value was used as a surrogate. The network meta-analysis was based on a random effects model, and correlation in multi-arm studies was considered [15]. The common heterogeneity variance ${\tau }^{2}$ used in the random effects model was estimated by a generalized DerSimonian-Laird estimator [16]. To assess inconsistency, the between-designs *Q* statistic was calculated based on a full design-by-treatment interaction random effects model [17]. The fitted models were used to compare the efficacy of different treatments, for two distinct time points and two separate outcomes.

The follow-up dates were grouped into two phases, the first describing treatments in the earlier phase, one to six months. If more than one follow-up date was specified, priority was given to 3 months, then 6 months, and 1 month as the last option. The second time point was considered the later phase, where 24 months, then 12 months, and as the final option, any follow-up dates beyond 24 months were prioritized.

The outcomes determining the efficacy of the treatment referred to the visual improvement measured by the BCVA in letters on the one hand and to the anatomical recovery measured by the CRT in micrometers on the other hand.

Furthermore, subgroup analysis for the different anti VEGFs was performed for the same time points and outcomes. In the primary analysis, we did not distinguish between one initial injection followed by a pro re nata approach (1 + PRN) and three initial injections followed by a PRN approach (3 + PRN); furthermore, we performed a separate pairwise meta-analysis to evaluate possible differences between the two treatment regimens. To compare the number of treatments, we used a two-sample *t*-test with Welch–Satterthwaite correction on pooled standard deviations and means.

A *p* value of less than 0.05 was considered statistically significant. All analyses were performed in R, Version 4.1.3 [18].

## Results

Our literature search yielded 1,156 articles (see Fig. 1). 166 full text articles of these were screened for eligibility. We included 64 studies for our qualitative and 34 studies for our quantitative analysis (see Tables 1 and 2).

**Table 1:** Table of study characteristic and inclusion criteria of all 64 studies included for qualitative analysis

ID_Study	Quantitative analysis	Randomized	*N*	Treatment	Country of origin	Definition of myopic CNV	Min. axial length	Inclusion criteria	Exclusion criteria	Pretreatment
Baba (2010) [27]	Yes	No	12	Bevacizumab	Japan	(i) < − 6dpt (ii) Type 2 juxta- and subfoveal CNV, active on FLA	n/d	(i) follow-up > 2 years (ii) initial onset of symptoms < 6 months	(i) BCVA < 0.1 at baseline (ii) Age < 40 years	None
			12	PDT						
Bandello (2003) [28]	No	No	12	PDT	Italy	(i) ≤ − 6dpt and/or (ii) AL ≥ 26, 5 mm	≥ 26, 5	(i) Active extrafoveal CNV on FA (ii) Previous treatment with laser photocoagulation (iii) Retinal abnormalities (iv) < 5,400 µm CNV dimension	(i) Other potential causes of CNV	Yes
			13	Untreated						
Bandello (2013) [29]	No	Yes	222	Ranibizumab	Multi-center	VIP study	n/d	(i) n/d	(i) n/d	n/d
			55	PDT						
Brancato (1988) [30]	No	Yes	9	Laser (577)	n/d	(i) < − 6dpt (ii) CNV documented with FLA < 7 days (iii) minimal distance of 100 microns from center of the foveal avascular zone	None	(i) BCVA ≥ 0.1	(i) Other ocular disease that could modify FLA	None
			9	Laser (590)						
			9	Laser (620)						
Brilliance Study [31]	No	Yes	182	Ranibizumab (VA guided)	Multi-center (5 countries)	(i) < − 6dpt (ii) AL ≥ 26 mm (iii) Myopic changes CNV leakage in FLA (iv) Intra-or subretinal fluid (v) Increase in central subfield thickness	≥ 26 mm	(i) BCVA ≥ 24 to ≤ 78	(i) nAMD (ii) Histoplasmosis (iii) Polypoidal choroidal vasculopathy (iv) Active infectious disease (v) Intraocular inflammation (vi) Infection (vii) Increased IOP (viii) RVO (ix) Diabetes mellitus (x) Severe DR (xi) Arterial hypertension (xii) Stroke or myocardial infarction within 3 months (xiii) PRP within 6 months (xiv) Focal macular laser at any time (xv) Anti-VEGF or PDT at any time (xvi) Intravitreal corticosteroids or surgery within 3 months (xvii) Pregnant women	None
			184	Ranibizumab (Disease guided)						
			91	PDT						
Calvo-González (2017) [32]	No	No	26	Ranibizumab (1 + PRN)	n/d	(i) < − 6dpt (ii) AL > 26.0 mm (iii) Retinal abnormalities (iv) Active sub- or juxtafoveal CNV	> 26 mm	(i) n/d	(i) PDT within 6 months (ii) Prior anti-VEGF treatment (iii) CNV due to other cause (iv) Previous thromboembolic episodes (v) Allergy to fluorescein (vi) Fertile women not using contraception (vii) Follow-up less than 24 months	Yes
			35	Ranibizumab(3 + PRN)						
Cha (2014) [33]	Yes	No	23	Ranibizumab	South Korea	(i) > 26 mm AL (ii) < − 6dpt (iii) Pathologic myope M2	> 26 mm	(i) No pretreatment (ii) BCVA 20/500–20/30 (iii) > 12 months follow-up	(i) History of intraocular surgery except cataract (ii) Cataract surgery < 6 months before enrollment (iii) Other ocular disorder decreasing visual acuity (iv) Cataract surgery or YAG capsulotomy during follow-up	None
			43	Bevacizumab						
Chan (2007) [34]	No	No	22	PDT + i.TCA	n/d	(i) ≤ − 6dpt (ii) Sub- or juxtafoveal CNV (iii) Leakage in FLA (iv) Greatest linear dimension < 5,400 µm	n/d	(i) BCVA ≥ 20/400	(i) CNV due to other causes (ii) Prior treatment (iii) History of glaucoma	None
			22	PDT						
Chen (2011) [35]	No	No	17	Bevacizumab	USA	Myopic CNV	n/d	(i) n/d	(i) n/d	n/d
			6	PDT + bevacizumab						
Chen (2020) [36]	Yes	No	31	Conbercept	China	n/d	> 26 mm	(i) BCVA > 20/800 (ii) > 18 years	(i) CNV secondary to other causes (ii) Other chorioretinopathies (iii) History of prior treatment	None
			33	Ranibizumab						
Costa (2006) [37]	No	Yes	8	PDT (standard 50 J/cm^2^)	Brazil	(i) < − 6dpt or AL ≥ 26 mm (ii) Retinal abnormalities (iii) CNV under foveal avascular zone	≥ 26.5 mm	(i) n/d	(i) Drusen (ii) Traumatic choroidal rupture (iii) Peripapillary changes with atrophic or pigmented “punched out” chorioretinal lesions (iv) Uveitis (v) Any other ophthalmic disorder that might affect visual function (vi) Disability to cooperate (vii) Allergy to fluorescein (viii) Porphyria (ix) Previous treatment for CNV (x) Significant opacities	None
			8	PDT (two-fold 100 J/cm^2^)						
Dethorey (2010) [38]	No	No	19	Ranibizumab	France	(i) ≤ − 6dpt or AL ≥ 26 mm (ii) Myopic CNV	≥ 26.5 mm	(i) n/d	(i) n/d	None
			34	PDT						
El Habbak (2016) [39]	No	Yes	10	Ranibizumab	Egypt	n/d	n/d	(i) n/d	(i) n/d	n/d
			10	Aflibercept						
Erden (2019) [40]	Yes	No	12	Aflibercept	Turkey	(i) < − 6dpt or AL > 26 mm (ii) Myopic CNV	> 26 mm	(i) n/d	(i) CNV due to other causes (ii) Uncontrolled glaucoma (iii) History of photocoagulation or PDT (iv) Iris neovascularization (v) Vitreous hemorrhage (vi) History of thromboembolic events	Yes
			18	Ranibizumab						
Farinha (2013) [41]	No	No	11	PDT	Portugal	(i) ≤ − 6dpt or AL ≥ 26 mm (ii) Myopic CNV	≥ 26 mm	(i) contralateral myopia without CNV (ii) Minimum follow-up of 3 years	(i) Amblyopia (ii) Glaucoma (iii) Uveitis (iv) Dense cataract (v) Diabetic retinopathy (vi) Retinal vascular abnormalities (vii) Laser treatment (viii) Intravitreal injection of triamcinolone (ix) Previous vitrectomy and scleral buckling	None
			8	Ranibizumab						
			9	PDT + ranibizumab						
Fernandez (2013) [42]	No	No	8	Ranibizumab	Spain	n/d	n/d	(i) Subfoveal	(i) n/d	n/d
			8	Bevacizumab						
Fonseca (2010) [43]	No	No	25	Bevacizumab	Portugal	n/d	n/d	(i) n/d	(i) n/d	n/d
			19	Ranibizumab						
Freitas-da-Costa (2014) [44]	No	No	67 (IVB + IVR)	Bevacizumab	Portugal	(i) < − 6dpt or less (ii) With retinal abnormalities or AL ≥ 26.5 mm (iii) CNV active disease with leakage in FLA	≥ 26.5	(i) Treatment with IVB (ii) IVR	(i) CNV secondary to other causes (ii) Retinal vascular disease (iii) Intraocular surgery during period of study	Yes
				Ranibizumab						
Gharbiya (2010) [45]	Yes	Yes	16	Ranibizumab	Italy	(i) AL > 26.5 mm (ii) CNV	> 26.5 mm	(i) Leakage from FLA	(i) Other ocular disease that could affect BCVA (ii) Angioid streaks (iii) Trauma (iv) Choroiditis (v) Hereditary diseases (vi) Aphakia (vii) Previous vitreoretinal surgery (viii) Prior history of bleeding diathesis (ix) Prior cerebrovascular accident (x) Pulmonary embolus or deep venous thrombosis (xi) Myocardial infarction (xii) Uncompensated CAD within 6 months (xiii) Major surgery within 6 weeks (xiv) uncontrolled hypertension	None
			16	Bevacizumab						
Glacet-Bernard (2007) [19]	Yes	No	34	PDT	France	(i) ≤ − 6dpt (ii) AL ≥ 26.5 mm	≥ 26.5 mm	(i) Subfoveal CNV (ii) BCVA 20/40 (iii) 20/100 for PDT (iv) ≤ 20/63 for translocation	(i) n/d	Yes
			32	Translocation						
Hamelin, 2002 [20]	No	No	18	Surgical Removal	France	n/d	n/d	i) subfoveal CNV	i) n/d	n/d
			14	Translocation						
Hayashi (2008) [46]	Yes	No	22	PDT	Japan	(i) ≤ − 6dpt (ii) AL ≥ 26.5 mm	≥ 26.5	(i) Greatest linear dimension of CNV lesion < 5,400 µm (ii) Active CNV (iii) FU > 6 months	(i) Other ocular disease such as large drusen (ii) Multifocal choroiditis (iii) Punctate inner choroidopathy (iv) Active hepatitis (v) Clinically significant liver disease (vi) Earlier treatment of CNV (vii) Porphyria (viii) Intraocular surgery within 2 months	None
			66	Untreated						
Hayashi (2009) [47]	Yes	No	43	Bevacizumab	Japan	(i) ≤ − 6dpt (ii) AL ≥ 26.5 mm	≥ 26.5	(i) FLA leakage from CNV (ii) FU > 1 year	n/d	Yes
			44	PDT						
			74	Untreated						
Howaidy (2019) [6]	Yes	Yes	24	Aflibercept	Egypt	(i) ≤ − 6dpt (ii) AL ≥ 26 mm (iii) Active CNV in FLA	≥ 26	(i) Patient complaint < 8 weeks (ii) Clear ocular media	(i) Previous vitreoretinal intervention (ii) Associated retinal disorders (e.g., angioid streaks and choroiditis) (iii) Coexisting macular pathology secondary to pathologic myopia (e.g., myopic tractional maculopathy and myopic macular hole) (iv) Myocardial infarction (v) Thromboembolic events < 6 months	None
			24	Ranibizumab						
Iacono (2012) [48]	Yes	Yes	23	Ranibizumab	Italy	(i) ≤ − 6dpt (ii) AL ≥ 26.5 mm	≥ 26.5 mm	(i) Baseline BCVA 20/32 (ii) 20/400 (iii) > 12 months (iv) post-menopause	(i) Intraocular surgery < 6 months (ii) Any other ocular disease that could compromise vision (iii) Ocular hypertension (iv) Glaucoma (v) Uncontrolled systemic hypertension (vi) Peripheral vascular disease (vii) History of thromboembolism (viii) Ischemic heart disease (ix) Stroke	None
			25	Bevacizumab						
Iacono (2017) [49]	Yes	No	15	Bevacizumab	Italy	(i) ≤ − 6dpt (ii) AL ≥ 26.5 mm	≥ 26.5 mm	(i) Sub- and juxtafoveal CNV (ii) FLA (iii) > 12 months (iv) Post-menopause (v) Fertile women using contraception	(i) Previous anti VEGF (ii) Intraocular surgery < 6 months (iii) Any other ocular surgery that could compromise vision in the study eye (iv) Pregnancy (v) Ocular hypertension (vi) Glaucoma (vii) Uncontrolled systemic hypertension (viii) Peripheral vascular disease (ix) History of thromboembolism (x) Stroke	None
			33	Ranibizumab						
Ikuno (2010) [50]	Yes	No	11	Bevacizumab	Japan	(i) ≤ − 6dpt (ii) AL ≥ 26.5 mm	≥ 26.5 mm	(i) Women (ii) > 50a (iii) Active sub- or juxtafoveal (iv) No history of pretreatment (v) Baseline BCVA 20/200–20/40 (vi) Baseline CNV size 1,200–3,000 µm	(i) History of vitrectomy (ii) Intraocular surgery other than cataract (iii) Presence of macular hole (iv) Retinal detachment (v) Foveoschisis (vi) Severe cataract (vii) Symptom duration > 24 months (viii) Significant glaucoma detected by visual field loss	None
			20	PDT						
Introini (2012) [51]	Yes	No	13	Bevacizumab	Italy	(i) < − 6dpt	n/d	(i) BCVA > 20/200	(i) Presence of retinal diseases (ii) Previous CNV treatment (iii) Intraocular surgery within the last 3 months (iv) Glaucoma (v) Pregnancy (vi) Uncontrolled systemic hypertension (vii) History of thromboembolic disease (viii) Ischemic cardiovascular disease	None
			9	Ranibizumab						
Kang (2017) [52]	Yes	No	17	Bevacizumab	Korea	n/d	n/d	(i) n/d	(i) n/d	n/d
			20	PDT						
Kobayashi (2000) [7]	No	Yes	20	Radiotherapy	Japan	(i) ≤ − 8dpt (ii) AL ≥ 26 mm	≥ 26 mm	(i) VA < 0.4, subfoveal (ii) Age > 60	(i) Other ocular disease such as glaucoma, chronic inflammation, or neoplastic disorder (ii) Systemic diseases (diabetes, uncontrolled hypertension, and known life-threatening disease)	n/d
			19	untreated						
Korol (2020) [53]	Yes	Yes	50	Ranibizumab	Ukraine and Arab Emirates	(i) ≤ − 6dpt (ii) AL ≥ 26.5 mm	≥ 26 mm	(i) New onset of myopic CNV < 2 months (ii) Age < 18a	(i) Other ocular disease (CNV, ocular inflammation, glaucoma, ocular hypertension, and opacity) (ii) Pregnancy (iii) Lactation (iv) Disability to provide informed consent	n/d
			47	Aflibercept						
Lai (2012) [54]	Yes	No	22	Bevacizumab	China	(i) < − 6dpt	n/d	(i) Follow-up > 2 years (ii) Subfoveal CNV (iii) BCVA > 20/800 (iv) FA leakage	(i) Prior treatment (ii) Secondary CNV to other ocular disease	None
			15	Ranibizumab						
Li (2019) [55]	No	Yes	26	Ranibizumab PRN + 1	China	(i) ≤ − 6dpt (ii) AL ≥ 26 mm	≥ 26 mm	(i) Active sub- or juxtafoveal CNV in FLA (ii) Baseline BCVA 24–73	(i) Presence of other ocular disease that affected VA (ii) Anti-VEGF within 6 months (iii) Previous PDT (iv) Intraocular surgery within 3 months (v) Uncontrolled glaucoma (vi) Pregnancy (vii) Severe systemic condition (uncontrolled hypertension, history of thromboembolic, or ischemic cardiovascular disease)	Yes
			24	RanibizumabPRN + 3						
Matsuo (2012) [56]	Yes	No	22	Anti-VEGF	Japan	(i) ≤ − 6dpt (ii) AL ≥ 26 mm	≥ 26 mm	(i) Active sub- or juxtafoveal CNV in FLA (ii) Visual symptoms (iii) Onset within 6 months (iv) Minimum follow-up 6 months	(i) History of RVO (ii) Uveitis (iii) Rhegmatogenous retinal detachment (iv) Glaucoma	None
			20	PDT						
Miki (2013) [21]	No	No	37	Anti-VEGF	Japan	(i) ≤ − 6dpt (ii) AL ≥ 26 mm	≥ 26 mm	(i) Subretinal lesions (ii) Hemorrhage		None
			20	PDT						
			21	Bisphosphonates						
			22	Untreated						
Myrror study [57]	No	Yes	90	Aflibercept	Japan	(i) ≤ − 6dpt (ii) AL ≥ 26 mm	≥ 26.5 mm	(i) Active CNV (ii) BCVA 73–35 letters	(i) 1 functional eye (ii) Recurrent myopic CNV (iii) Aphakia (iv) History of CNV with other origin (v) Ocular inflammation (vi) NVI (vii) Vitreous hemorrhage (viii) Uncontrolled glaucoma (ix) Previous filtration surgery (x) Pregnant women (xi) Breast-feeding women	None
			31	Sham/placebo						
Ng (2015) [14]	No	No	77	Bevacizumab (3 + PRN)	China	(i) ≤ − 6.0 diopters	n/d	(i) Follow-up > 1 year (ii) Evidence of leakage on FA	(i) PDT or triamcinolone during follow-up (ii) CNV secondary to AMD or other causes such as trauma, choroiditis, angioid streaks, and hereditary disease (iii) Cataract or refractive surgery during follow-up (iv) History of vitrectomy (v) Serious posterior segment complications such as retinal detachment or foveoschisis (vi) History of previous anti-VEGF treatment	Yes
			16	Bevacizumab (1 + PRN)						
Niwa (2012) [58]	No	No	13	Bevacizumab (1 + PRN)	Japan	(i) ≤ − 6dpt (ii) AL ≥ 26 mm	≥ 26.5 mm	(i) n/d	(i) Other causes of CNV (ii) Previous treatment	None
			19	Bevacizumab (3 + PRN)						
Pal (2010) [59]	No	No	22	Untreated	London	n/d	n/d	n/d	(i) n/d	n/d
			8	PDT						
			21	Anti-VEGF						
Parodi (2010) [22]	Yes	Yes	18	PDT	Italy	(i) ≤ − 6dpt (ii) AL ≥ 26 mm (iii) Retinal abnormalities	≥ 26 mm	(i) Juxtafoveal CNV on FA (ii) > 5,400 µm CNV size (iii) BCVA 20/200 to 20/40 (iv) Symptoms < 1 month (v) Documented visual acuity deterioration	(i) Any other condition associated with CNV (ii) Any significant ocular disease that could compromise vision (iii) Active hepatitis (iv) Clinically significant liver disease (v) Peripheral vascular disease (vi) Thromboembolism (vii) Stroke (viii) Intraocular surgery < 2 months (ix) Pervious laser photocoagulation	n/d
			17	Krypton laser photocoagulation						
			19	Bevacizumab						
Parravano (2014) [60]	Yes	No	43	PDT	Italy	(i) ≤ − 6dpt	n/d	(i) Follow-up > 1 year		None
			42	Ranibizumab						
Pece (2015) [61]	Yes	Yes	40	Bevacizumab	Italy	(i) ≤ − 6dpt	n/d	(i) Myopic retinal changes of posterior pole (ii) FA active CNV (iii) BCVA > 20/400 at baseline (iv) Duration of symptoms < 4 weeks (v) Clear ocular media	(i) Retinal disease other than myopia (ii) Extrafoveal CNV (iii) Other chorioretinal alterations (iv) Refractive media opacities (v) Recent myocardial infarction (vi) Other thromboembolic events (vii) Previous intravitreal injections	None
			38	Ranibizumab						
Radiance [62]	No	Yes	106	Ranibizumab (VA guided)	International	(i) ≤ − 6dpt (ii) AL ≥ 26 mm	≥ 26 mm	(i) Active leakage from CNV (ii) Presence of retinal or subretinal fluid (iii) Increase in retinal thickness (iv) BCVA 24–78	(i) History of stroke (ii) History of retinal or focal laser photocoagulation (iii) Intraocular treatment with corticosteroid (iv) Surgery within prior 3 months (v) Hypersensitivity to ranibizumab (vi) CNV secondary to other causes (vii) Active infectious disease (viii) Intraocular inflammation (ix) IOP > 25 mmHg (x) Iris neovascularization (xi) Pregnant or nursing women	None
			116	Ranibizumab (disease guided)						
			55	PDT						
Rinaldi (2017) [63]	Yes	Yes	20	PDT	Italy	(i) ≤ − 6dpt (ii) AL ≥ 26 mm (iii) Retinal abnormalities	≥ 26 mm	(i) FA sub- or juxtafoveal CNV (ii) Clear ocular media (iii) Duration of (iv) Symptoms < 4 weeks	(i) Prior treatment (ii) Presence of another maculopathy (iii) History of myocardial infarction (iv) Other thromboembolic event (v) Uncontrolled hypertension (vi) Uncontrolled glaucoma (vii) Refractive media opacities (viii) Ocular surgery	None
			20	PDT + ranibizumab						
			20	Ranibizumab						
Rishi (2011) [64]	No	No	11	PDT	India	(i) ≤ − 6dpt	n/d	(i) Active CNV on FA	(i) n/d	n/d
			3	PDT + i.TCA						
			5	PDT + bevacizumab						
			4	PDT + ranibizumab						
			3	PDT + ranibizumab (reduced fluence)						
Rishi (2016) [65]	Yes	No	23	PDT	India	(i) ≤ − 6dpt	n/d	(i) n/d	(i) n/d	n/d
			25	Anti-VEGF						
			31	PDT + anti-VEGF						
Ruiz-Moreno (2011a) [66]	Yes	Yes	28	PDT	Spain	(i) ≤ − 6dpt (ii) AL ≥ 26 mm	≥ 26 mm	(i) < 18a (ii) Active sub- and juxtafoveal CNV (iii) Decreased VA (iv) Attributable to CNV	(i) Previous vitrectomy (ii) Tractional maculopathy (iii) Pregnant women (iv) Fertile women not willing to use contraception	n/d
			27	Bevacizumab						
Ruiz-Moreno (2011b) [67]	Yes	No	19	Bevacizumab (3 + PRN)	Spain	n/d	n/d	(i) n/d	(i) n/d	
			20	Bevacizumab (1 + PRN)						
Ruiz-Moreno (2012) [68]	No	No	107	Bevacizumab (1 + PRN)	Spain and Portugal	n/d	n/d	(i) n/d	(i) n/d	Yes
			32	Bevacizumab (3 + PRN)						
Ruiz-Moreno (2013a) [69]	Yes	No	53	Bevacizumab	Spain and Portugal	(i) ≤ − 6dpt (ii) AL ≥ 26 mm	≥ 26 mm	(i) n/d	(i) Retinal drusen (ii) AMD	Yes
			24	Ranibizumab						
Ruiz-Moreno (2013b) [70]	Yes	Yes	28	PDT	Spain	(i) ≤ − 6dpt (ii) AL ≥ 26 mm	≥ 26 mm	(i) < 18a (ii) Active sub- or juxtafoveal CNV (iii) Decreased VA attributable to CNV	(i) Previous vitrectomy (ii) Tractional maculopathy (iii) Pregnant women (iv) Fertile women not willing to use contraception	n/d
			27	Bevacizumab						
Ruiz-Moreno (2015) [71]	Yes	No	78	Bevacizumab	Spain and Portugal	(i) ≤ − 6dpt (ii) AL ≥ 26 mm (iii) Fundus changes of high myopia	≥ 26 mm	(i) n/d	(i) Less than 6-year follow-up (ii) Retinal drusen (iii) AMD (iv) Previously vitrectomized (v) Treated for mCNV with two or more intravitreal drugs or PDT	Yes
			19	Ranibizumab						
Saviano (2014) [72]	Yes	No	17	PDT + bevacizumab	Italy	(i) ≤ − 6dpt (ii) AL ≥ 26 mm	≥ 25 mm	(i) n/d	(i) Membranes correlated to pathologic myopia (ii) Glaucoma (iii) Intolerance to medication used	Yes
			17	Bevacizumab						
Sayanagi (2019) [73]	Yes	No	12	Ranibizumab	Japan	(i) ≤ − 6dpt (ii) AL ≥ 26 mm	≥ 26.5 mm	(i) Sub- or juxtafoveal CNV	(i) Treatment other than anti-VEGF before or during observation (ii) Follow-up < 6 months (iii) Intraocular surgery other than cataract surgery (iv) Other ocular diseases during follow-up	None
			15	Aflibercept						
Siu-Chun (2015) [74]	No	No	77	Bevacizumab (3 + PRN)	China	(i) ≤ − 6dpt	n/d	(i) FU > 1 year (ii) CNV leakage on FA	(i) History of PDT or subtenon or intravitreal triamcinolone within 3 months (ii) CNV due to other causes in the study or fellow eyes (iii) Cataract extraction or refractive surgery after IVB (iv) History of vitrectomy (v) Presence of serious posterior segment (vi) History of previous anti-VEGF treatment in another institute	Yes
			16	Bevacizumab (1 + PRN)						
VIP-Blinder (2001) [75]	No	Yes	81	PDT	Multi-center	(i) < − 6dpt or less (ii) With retinal abnormalities (iii) AL > 26.5 mm	> 26.5 mm	(i) CNV under FAZ (ii) CNV > 50% of total neovascular lesion (iii) < 5,400 µm CNV size (iv) BCVA ≥ 50	(i) Any other condition associated with CNV (ii) RPE tear (iii) Any ocular disease compromising vision (iv) History of CNV other than no foveal confluent laser photocoagulation (v) Prior PDT (vi) IOL surgery within last 2 months (vii) Active hepatitis (viii) Porphyria (ix) Participation in other clinical trial (x) Pregnancy	Yes
			39	Sham/placebo						
VIP1 Arnold (2001) [76]	No	Yes	81	PDT	Multi-center	(i) < − 6dpt or less (ii) With retinal abnormalities (iii) AL > 26.5 mm	> 26.5 mm	(i) CNV under FAZ (ii) CNV > 50% of total neovascular lesion (iii) < 5,400 µm CNV size (iv) BCVA ≥ 50	(i) Any other condition associated with CNV (ii) RPE tear (iii) Any ocular disease compromising vision (iv) History of CNV other than no foveal confluent laser photocoagulation (v) Prior PDT (vi) IOL surgery within last 2 months (vii) Active hepatitis (viii) Porphyria (ix) Participation in other clinical trial (x) Pregnancy	Yes
			39	Sham/placebo						
Voykov (2010) [77]	Yes	No	11	Bevacizumab	Germany	(i) ≤ − 6dpt	n/d	(i) Sub- or juxtafoveal CNV (ii) BCVA > 20/400	(i) CNV secondary to other causes in study or fellow eye	Yes
			10	PDT + bevacizumab						
Wakabayashi (2009) [23]	Yes	No	20	Subtenon TCA	Japan	(i) ≤ − 6dpt	n/d	(i) Active CNV on FA	(i) Extrafoveal CNV (ii) BCVA < 20/200 (iii) Previous treatment such as PDT or photocoagulation (iv) History of cataract (v) Vitreous surgery	None
			34	Bevacizumab						
Wakabayashi (2011) [78]	No	No	19	Bevacizumab (1 + PRN)	Japan	(i) ≤ − 6dpt (ii) AL ≥ 26 mm	≥ 26.5 mm	(i) Newly developed and active mCNV	(i) < 20/200 BCVA (ii) History of scleral buckling (iii) Vitreous surgery (iv) Other treatments such as photodynamic therapy and photocoagulation	None
			12	Bevacizumab (3 + PRN)						
Wang (2018) [79]	Yes	No	36	Aflibercept	Taiwan	(i) > 26 mmHg	≥ 26 mm	(i) Treatment-naïve (ii) > 18a (iii) BCVA 20/400–20/40	(i) Pregnant (ii) Nursing (iii) History of thromboembolic events (iv) Major surgery within previous 3 months (v) Uncontrolled hypertension (vi) Known coagulation abnormalities (v) Use of anticoagulants other than aspirin (vi) Prior macular photocoagulation or PDT (vii) Prior intraocular surgery within 3 months (viii) Active infectious disease or inflammation (ix) Intraocular pressure > 25 mmHg (x) Presence of iris neovascularization (xi) Vitreous hemorrhage	None
			42	Bevacizumab						
Woronkowicz (2018) [80]	No	No	85	Bevacizumab	United Kingdom	n/d	n/d	(i) n/d	(i) n/d	n/d
			125	Ranibizumab						
Yoon (2010) [81]	Yes	No	51	PDT	Korea	(i) ≤ − 6dpt (ii) AL ≥ 26 mm	≥ 26.5	(i) Active CNV on FLA (ii) BCVA > 20/400 (iii) Follow-up > 12 months	(i) Prior laser photocoagulation on study eye (ii) Radiation on study eye (iii) Vitrectomy on study eye (iv) History of subtenon injection of triamcinolone acetonide (v) PDT or anti-VEGF within 6 months (vi) Cataract surgery during follow-up (vii) Presence of comorbid ocular conditions	Yes
			63	Anti-VEGF						
			28	PDT + anti VEGF						
Yoon (2012) [82]	Yes	No	14	Ranibizumab	Korea	(i) ≤ − 6dpt (ii) AL ≥ 26 mm	≥ 26.5	(i) Active CNV on FLA (ii) BCVA > 20/400 (iii) Follow-up > 12 months (iv) Sub- or juxtafoveal CNV	(i) History of previous treatment (ii) Cataract surgery within follow-up period (iii) Presence of comorbid ocular conditions that might affect VA	Yes
			26	Bevacizumab						

*AEs*, adverse events; *AL*, axial length; *AMD*, age-related macular degeneration; *Anti-VEGF*, anti-vascular endothelial growth factor; *BCVA*, best-corrected visual acuity; *CNV*, choroidal neovascularization; *DR*, diabetic retinopathy; *FA/FLA*, fluoresceine angiography; *FAZ*, foveal avascular zone; *FU*, follow-up; *iTCA*, intravitreal triamcinolone; *IOL*, intraocular lens; *IOP*, intraocular pressure; *IVB*, intravitreal bevacizumab; *IVR*, intravitreal ranibizumab; *N*, number of eyes; *n/d*, non-defined; *nAMD*, neovascular age-related macular degeneration; *PDT*, photodynamic therapy; *PRN*, pro re nata; *PRP*, panretinal photocoagulation; *RPE*, retinal pigment epithelium; *RVO*, retinal vein occlusion; *SAEs*, severe adverse events; *VA*, visual acuity

**Table 2:** Table of complications rates for all 64 studies included for qualitative analysis

ID_Study	Quantitative analysis	*N*^a^	Treatment	Ocular complications	Other	Anti-VEGF treatment
Baba (2010) [27]		12	Bevacizumab	0 (0%)		
	Yes	12	PDT	0 (0%)		
Bandello (2003) [28]	No	12	PDT	0 (0%)		
		13	Untreated	0 (0%)		
Bandello, 2013 [29]	No	222	Ranibizumab	2 (0,8%) SAEs (corneal erosion)	11 (4.9%) SAEs (i) Myocarditis (ii) Atrial tachycardia (iii) Lung adenocarcinoma (iv) Subdural hematoma	1 + PRN (VA stability versus Disease activity)
		55	PDT	0 (0%) SAEs	0 (0%) SAEs	
Brancato (1988) [30]	No	9	Laser (577)	n/d	n/d	
		9	Laser (590)	n/d	n/d	
		9	Laser (620)	n/d	n/d	
Brilliance Study [31]	No	182	Ranibizumab (VA guided)	1 (< 1%) retinal detachment	0 (0%)	2 + PRN visual acuity guided
		184	Ranibizumab (disease guided)	1 (< 1%) retinal detachment	0 (0%)	1 + PRN disease guided
		91	PDT	1 (< 1%) (1 endophthalmitis after switch to ranibizumab)	0 (0%)	
Calvo-González (2017) [32]	No	26	Ranibizumab	n/d	n/d	1 + PRN
		35	Ranibizumab	n/d	n/d	3 + PRN
Cha (2014) [33]	Yes	23	Ranibizumab	0 (0%)	0 (0%)	1 + PRN
		43	Bevacizumab	0 (0%)	0 (0%)	1 + PRN
Chan (2007) [34]	No	22	PDT + i.TCA	10 (46%) IOP increase 3 (20%) cataract progression	0 (0%)	
		22	PDT	0 (0%)	0 (0%)	
Chen (2011) [35]	No	17	Bevacizumab	n/d	n/d	
		6	PDT + Bevacizumab	n/d	n/d	
Chen (2020) [36]	Yes	31	Conbercept	0 (0%)	0 (0%)	1 + PRN
		33	Ranibizumab	0 (0%)	0 (0%)	1 + PRN
Costa (2006) [37]	No	8	PDT (standard 50 J/cm^2^)	n/d	n/d	
		8	PDT (two-fold 100 J/cm^2^)	n/d	n/d	
Dethorey (2010) [38]	No	19	Ranibizumab	n/d	n/d	
		34	PDT	n/d	n/d	
El Habbak (2016) [39]	No	10	Ranibizumab	n/d	n/d	1 + PRN
		10	Aflibercept	n/d	n/d	1 + PRN
Erden (2019) [40]	Yes	12	Aflibercept	0 (0%)	0 (0%)	1 + PRN
		18	Ranibizumab	0 (0%)	0 (0%)	1 + PRN
Farinha (2013) [41]	No	11	PDT	n/d	n/d	
		8	Ranibizumab	n/d	n/d	
		9	PDT + ranibizumab	n/d	n/d	PDT + IVR not simultaneous but rather patients with PDT were switched to IVR if deemed necessary
Fernandez (2013) [42]	No	8	Ranibizumab	0 (0%)	0 (0%)	1 + PRN
		8	Bevacizumab	0 (0%)	0 (0%)	1 + PRN
Fonseca (2010) [43]	No	25	Bevacizumab	0 (0%)	0 (0%)	1 + PRN
		19	Ranibizumab	0 (0%)	0 (0%)	1 + PRN
Freitas-da-Costa (2014) [44]	No	67 (IVB + IVR)	Bevacizumab	1 (< 1%) sterile vitritis	0 (0%)	1 + PRN
			Ranibizumab	0 (0%)	0 (0%)	1 + PRN
Gharbiya (2010) [45]	Yes	16	Ranibizumab	0 (0%)	0 (0%)	1 + PRN
		16	Bevacizumab	0 (0%)	0 (0%)	1 + PRN
Glacet-Bernard (2007) [19]	Yes	34	PDT	0 (0%)	0 (0%)	
		32	Translocation	3 (9.3%) retinal detachment 1 (3%) macular hole 1 (3%) macular fold 2 (6%) transitory diplopia 2 (6%) diplopia treated with prism 10 (23%) cataract extraction	0 (0%)	
Hamelin (2002) [20]	No	18	Surgical removal	7 (39%) CNV recurrence 2 (11%) retinal detachment 1 (5%) subretinal hemorrhage	0 (0%)	
		14	Translocation	2 (14%) CNV recurrence 2 (14%) retinal detachment 1 (7%) hyphemia 1 (7%) macular hole 2 (14%) transient diplopia	0 (0%)	
Hayashi (2008) [46]	Yes	22	PDT	2 (9%) occlusions of large choroidal vessels	0 (0%)	
		66	Untreated	0 (0%)	0 (0%)	
Hayashi (2009) [47]	Yes	43	Bevacizumab	0 (0%)	n/d	
		44	PDT	n/d	n/d	
		74	untreated	n/d	n/d	
Howaidy (2019) [6]	Yes	24	Aflibercept	0 (0%)	0 (0%)	3 + PRN
		24	Ranibizumab	0 (0%)	0 (0%)	3 + PRN
Iacono (2012) [48]	Yes	23	Ranibizumab	0 (0%)	0 (0%)	1 + PRN
		25	Bevacizumab	0 (0%)	0 (0%)	1 + PRN
Iacono (2017) [49]	Yes	15	Bevacizumab	0 (0%)	0 (0%)	1 + PRN
		33	Ranibizumab	0 (0%)	0 (0%)	1 + PRN
Ikuno (2010) [50]	Yes	11	Bevacizumab	0 (0%)	0 (0%)	1 + PRN
		20	PDT	1 (5%)	n/d	1 + PRN
Introini (2012) [51]	Yes	13	Bevacizumab	0 (0%)	0 (0%)	1 + PRN
		9	Ranibizumab	0 (0%)	0 (0%)	1 + PRN
Kang (2017) [52]	Yes	17	Bevacizumab	n/d	n/d	
		20	PDT	n/d	n/d	
Kobayashi (2000) [7]	No	20	Radiotherapy	1 (5%) conjunctival irritation	0 (0%)	
		19	Untreated	0 (0%)	0 (0%)	
Korol (2020) [53]	Yes	50	Ranibizumab	0 (0%)	0 (0%)	2 + PRN
		47	Aflibercept	0 (0%)	0 (0%)	2 + PRN
Lai (2012) [54]	Yes	22	Bevacizumab	2 (9%) cataract progression 1 (4.5%) increase in myopic foveoschisis 1 (4.5%) macular hole 1 (4.5%) retinal detachment	0 (0%)	3 + PRN
		15	Ranibizumab	1 (7%) cataract progression 1 (7%) progression in myopic foveoschisis 1 (7%) cellophane maculopathy 1 (7%) retinal thinning	0 (0%)	3 + PRN
Li (2019) [55]	No	26	Ranibizumab	0 (0%)	0 (0%)	1 + PRN
		24	Ranibizumab	1 (4%) retinal detachment	0 (0%)	3 + PRN
Matsuo (2012) [56]	Yes	22	Anti-VEGF	n/d	n/d	1 + PRN
		20	PDT	n/d	n/d	
Miki (2013) [21]	No	37	Anti-VEGF	0 (0%)	0 (0%)	1 + PRN
		20	PDT	0 (0%)	0 (0%)	
		21	Bisphosphonates	0 (0%)	0 (0%)	
		22	Untreated	0 (0%)	0 (0%)	
Myrror study [57]	No	90	Aflibercept	1 (1%) SAE macular hole	1 (1%) thromboembolic event	1 + PRN
		31	Sham/placebo	0 (0%)	0 (0%)	
Ng (2015) [14]	No	77	Bevacizumab	n/d	n/d	3 + PRN
		16	Bevacizumab	n/d	n/d	1 + PRN
Niwa (2012) [58]	No	13	Bevacizumab	0 (0%)	0 (0%)	1 + PRN
		19	Bevacizumab	0 (0%)	0 (0%)	3 + PRN
Pal (2010) [59]	No	22	Untreated	n/d	n/d	
		8	PDT	n/d	n/d	
		21	Anti-VEGF	n/d	n/d	
Parodi (2010) [22]	Yes	18	PDT	0 (0%)	0 (0%)	
		17	Krypton laser photocoagulation	0 (0%)	0 (0%)	
		19	Bevacizumab	0 (0%)	0 (0%)	1 + PRN
Parravano (2014) [60]	Yes	43	PDT	n/d	n/d	
		42	Ranibizumab	n/d	n/d	1 + PRN
Pece (2015) [61]	Yes	40	Bevacizumab	0 (0%)	0 (0%)	1 + PRN
		38	Ranibizumab	2 (5%) mild anterior Tyndall the day after the first injection	0 (0%)	1 + PRN
Radiance [62]	No	106	Ranibizumab	1 (< 1%) corneal erosion 12 (11.3%) conjunctival hemorrhage 8 (7.5%) punctate keratitis 4 (3.7%) dry eyes 4 (3.7%) eye pain 3 (2.8%) injection site hemorrhage 3 (2.8%) increased IOP 1 (< 1%) cataract (12 months)	0 (0%)	VA guided
		116	Ranibizumab	1 (< 1%) retinoschisis 12 (10%) conjunctival hemorrhage 3 (2.5%) punctate keratitis 2 (1.7%) dry eyes 4 (3.4%) eye pain 3 (2.5%) injection site hemorrhage 7 (6%) increased IOP 2 (1.7%) cataracts (12 months)	0 (0%)	Disease guided
		55	PDT	1 (1.8%) dry eye 1 (1.8%) eye pain 1 (1.8%) cataract (3 months)	0 (0%)	
Rinaldi (2017) [63]	Yes	20	PDT	0 (0%)	0 (0%)	
		20	PDT + ranibizumab	0 (0%)	0 (0%)	PDT + 1 + PRN
		20	Ranibizumab	0 (0%)	0 (0%)	3 + PRN
Rishi (2011) [64]	No	11	PDT	0 (0%)	0 (0%)	
		3	PDT + i.TCA	0 (0%)	0 (0%)	
		5	PDT + bevacizumab	0 (0%)	0 (0%)	
		4	PDT + ranibizumab	0 (0%)	0 (0%)	
		3	PDT + ranibizumab (reduced fluence)	0 (0%)	0 (0%)	
Rishi (2016) [65]	Yes	23	PDT	3 (13%) chorioretinal atrophy	0 (0%)	
		25	Anti-VEGF	0 (0%)	0 (0%)	
		31	PDT + anti-VEGF	2 (6.5%) chorioretinal atrophy	0 (0%)	
Ruiz-Moreno (2011a) [66]	Yes	28	PDT	0 (0%)	0 (0%)	
		27	Bevacizumab	0 (0%)	0 (0%)	3 + PRN
Ruiz-Moreno (2011b) [67]	Yes	19	Bevacizumab	0 (0%)	0 (0%)	3 + PRN
		20	Bevacizumab	0 (0%)	0 (0%)	1 + PRN
Ruiz-Moreno (2012) [68]	No	107	Bevacizumab	n/d	n/d	1 + PRN
		32	Bevacizumab	n/d	n/d	3 + PRN
Ruiz-Moreno (2013a) [69]	Yes	53	Bevacizumab	2 lens opacities (not attributed to one group)	0 (0%)	1 + and 3 + PRN
		24	Ranibizumab		0 (0%)	
Ruiz-Moreno (2013b) [70]	Yes	28	PDT	0 (0%)	0 (0%)	
		27	Bevacizumab	0 (0%)	0 (0%)	3 + PRN
Ruiz-Moreno (2015) [71]	Yes	78	Bevacizumab	2 lens opacities (not attributed to one group)	0 (0%)	1 + and 3 + PRN
		19	Ranibizumab		0 (0%)	
Saviano (2014) [72]	Yes	17	PDT + bevacizumab	0 (0%)	0 (0%)	1 + PRN + PDT
		17	Bevacizumab	0 (0%)	0 (0%)	3 + PRN
Sayanagi (2019) [73]	Yes	12	Ranibizumab	n/d	n/d	1 + PRN
		15	Aflibercept	n/d	n/d	1 + PRN
Siu-Chun (2015) [74]	No	77	Bevacizumab	0 (0%)	0 (0%)	3 + PRN
		16	Bevacizumab	0 (0%)	0 (0%)	1 + PRN
VIP-Blinder (2001) [75]	No	81	PDT	59 (73%) AEs	59 (73%) AEs	
		39	Sham/placebo	27 (69%) AEs	27 (69%) AEs	
VIP1 Arnold (2001) [76]	No	81	PDT	n/d	n/d	
		39	Sham/placebo	n/d	n/d	
Voykov (2010) [77]	Yes	11	Bevacizumab	0 (0%)	0 (0%)	1 + PRN
		10	PDT + bevacizumab	0 (0%)	0 (0%)	1 + PRN
Wakabayashi (2009) [23]	Yes	20	Subtenon TCA	3 (15%) IOP > 21 mmHg	0 (0%)	
		34	Bevacizumab	0 (0%)	0 (0%)	
Wakabayashi (2011) [78]	No	19	Bevacizumab	0 (0%)	0 (0%)	1 + PRN
		12	Bevacizumab	0 (0%)	0 (0%)	3 + PRN
Wang (2018) [79]	Yes	36	Aflibercept	0 (0%)	0 (0%)	1 + PRN
		42	Bevacizumab	0 (0%)	0 (0%)	1 + PRN
Woronkowicz (2018) [80]	No	85	Bevacizumab	n/d	n/d	
		125	Ranibizumab	n/d	n/d	
Yoon (2010) [81]	Yes	51	PDT	0 (0%)	0 (0%)	1 + PRN
		63	Anti-VEGF	0 (0%)	0 (0%)	1 + PRN
		28	PDT + anti-VEGF	0 (0%)	0 (0%)	1 + PRN
Yoon (2012) [82]	Yes	14	Ranibizumab	0 (0%)	0 (0%)	1 + PRN
		26	Bevacizumab	0 (0%)	0 (0%)	1 + PRN

*AEs*, adverse events; *AMD*, age-related macular degeneration; *Anti-VEGF*, anti-vascular endothelial growth factor; *CNV*, choroidal neovascularization; *FLA*, fluoresceine angiography; *i.TCA*, intravitreal triamcinolone; *IOP*, intraocular pressure; *IVB*, intravitreal bevacizumab; *IVR*, intravitreal ranibizumab; *N*, number of eyes; *N/d*, non-defined;^.^ *nAMD*, neovascular age-related macular degeneration; *PDT*, photodynamic therapy; *PRN*, pro re nata; *RVO*, retinal vein occlusion; *SAEs*, serious adverse events; *VA*, visual acuity

### Study characteristics

In the quantitative analysis, we included 34 studies comprising 2,098 eyes from 2,059 patients. 29 studies had two arms and 5 three arms. In the qualitative analysis comprising 64 studies and 4,641 eyes, 52 were two-arm studies, 9 three-arm studies, one was a four-arm study, and 2 were five-arm studies.

### Outcome in the earlier phase (≤ 6 months)

**Fig. 2:**
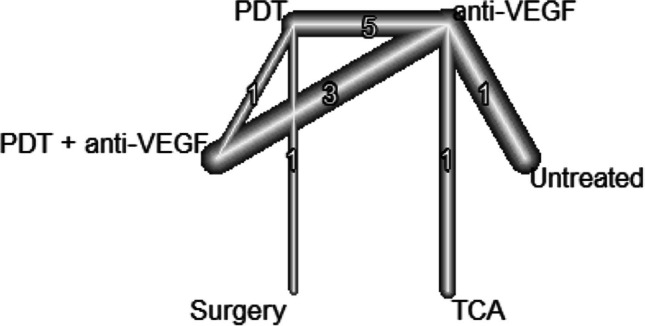
The structure of the network comparing different treatments regarding BCVA in the early phase (< 6 month). The numbers represent the numbers of direct comparisons, while the thickness of the lines is proportional to the inverse standard error of the estimates. BCVA, best-corrected visual acuity; PDT, photodynamic treatment; TCA, intravitreal triamcinolone acetonide; VEGF, vascular endothelial growth factors

The evidence network for BCVA in the early phase included 10 studies, representing 5 treatments and no treatment (see Fig. 2).

**Fig. 3:**
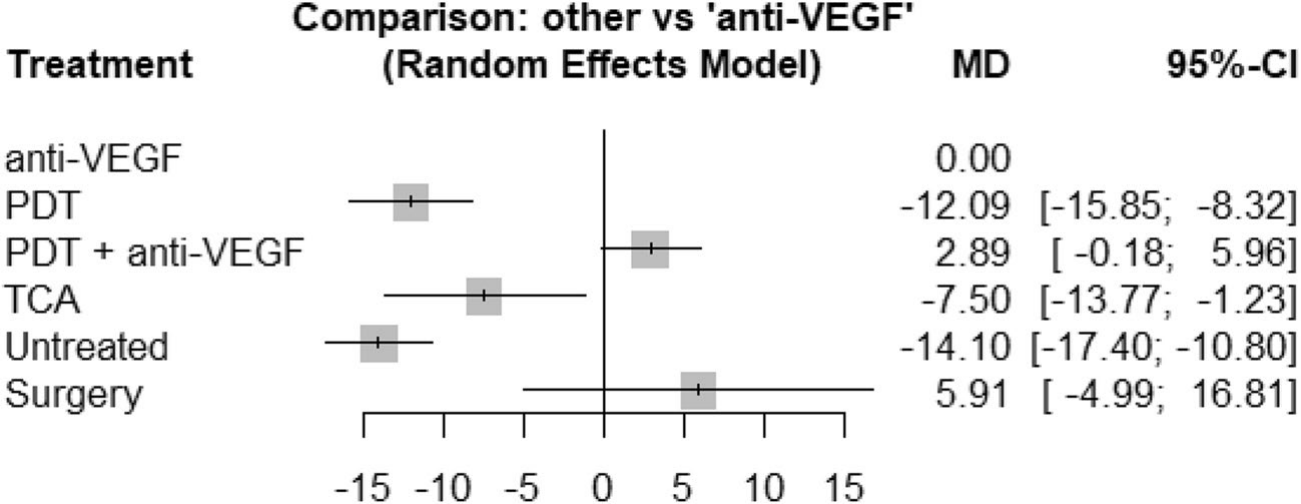
Forrest plot comparing change in BCVA (letters) before six months in the anti-VEGF treatment group compared to the other treatment groups. CI, confidence interval; MD, mean difference; PDT, photodynamic treatment; TCA, intravitreal triamcinolone acetonide; VEGF, vascular endothelial growth factors

In the early phase (≤ 6 months), patients treated by anti-VEGF gained on average 14.1 letters (95% CI, 10.8–17.4) more compared to untreated patients (*p* < 0.0001). Likewise, patients treated by anti-VEGF gained on average 12.1 letters (95% CI, 8.3–15.8) more than patients treated by PDT (*p* < 0.0001) and 7.5 letters (95% CI, 1.2–13.8) more than patients treated by intravitreal triamcinolone acetonide (TCA) (*p* = 0.019). The combination of PDT and anti-VEGF did not result in better visual outcome (MD − 2.9; 95% CI, − 6.0–0.2; *p* = 0.065) (see Fig. 3).

The other treatment modalities showed less favorable results in the early phase (≤ 6 months). Patients treated with TCA had gained in the mean 6.6 letters (95% CI, − 0.5–13.7) more compared to untreated patients (*p* = 0.068). The PDT treatment group had no significant change in visual acuity compared to the untreated group (MD − 2.01 letters; 95% CI, − 7.0 − 3.0; *p* = 0.430). There was no evidence of inconsistency within the network (*p* = 0.204).

**Fig. 4:**
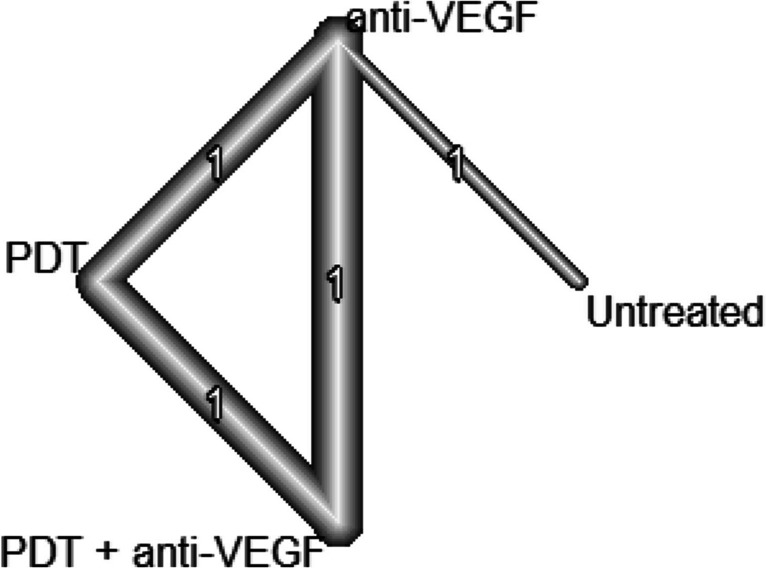
The structure of the network comparing different treatments regarding BCVA in the early phase (< 6 month). The numbers represent the numbers of direct comparisons, while the thickness of the lines is proportional to the inverse standard error of the estimates. PDT, photodynamic treatment; TCA, intravitreal triamcinolone acetonide; VEGF, vascular endothelial growth factors

**Fig. 5:**
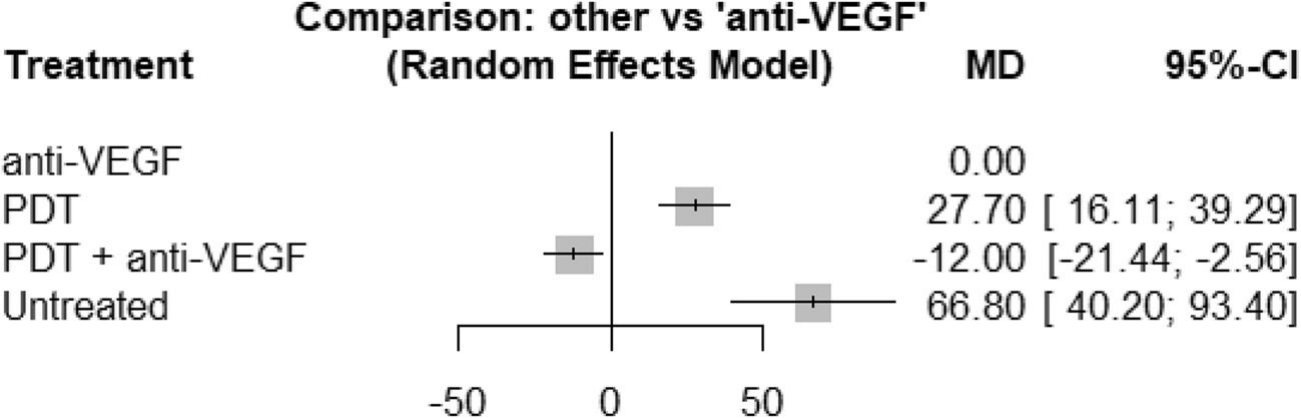
Forrest plot comparing change in central retinal thickness before six months in the anti-VEGF treatment group compared to the other treatment groups. CI, confidence interval; MD, mean difference; PDT, photodynamic treatment; VEGF, vascular endothelial growth factors

For central retinal thickness (CRT) in the early phase, only 2 studies were included (one two arm and one three arm study). The resulting network structure is therefore very simple (see Fig. 4). Even though the number of comparisons is small, the fitted network meta-analysis shows similar results compared to the analysis of BCVA. We can observe a significant decrease in CRT in patients treated with anti-VEGF compared to untreated patients (66.8 μm; 95% CI, 40.2 − 93.4; *p* < 0.0001) and patients treated with PDT (27.7 μm; 95% CI, 16.1–39.3; *p* < 0.0001). The combination treatment of PDT and anti VEGF therapy had a significant larger decrease in CRT than patients treated solely with anti-VEGF (12.0 μm; 95% CI, 21.4–2.6; *p* = 0.013) (see Fig. 5). Due to the small number of included studies, it is not reasonable to assess inconsistency.

Patients treated with 1 + PRN anti-VEGF gained 0.8 letters less (95% CI, − 2.8–4.5; *p* = 0.652) and their CRT decreased 20.0 μm less (95% CI, − 44.7–4.6; *p* = 0.111) compared to patients treated with 3 + PRN.

### Outcome in the later phase (> 6 months)

**Fig. 6:**
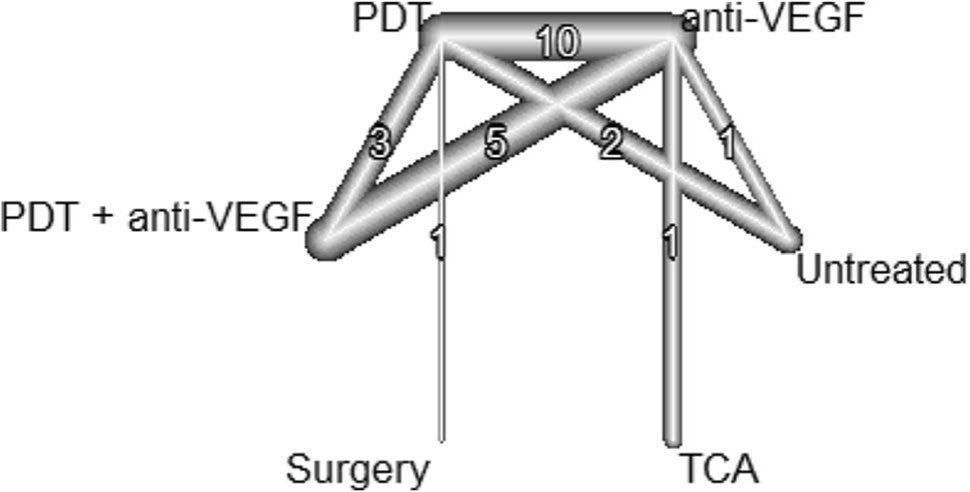
The structure of the network comparing different treatments regarding BCVA in the early phase (< 6 month). The numbers represent the numbers of direct comparisons, while the thickness of the lines is proportional to the inverse standard error of the estimates. BCVA, best-corrected visual acuity; PDT, photodynamic treatment; TCA, intravitreal triamcinolone acetonide; VEGF, vascular endothelial growth factors

**Fig. 7:**
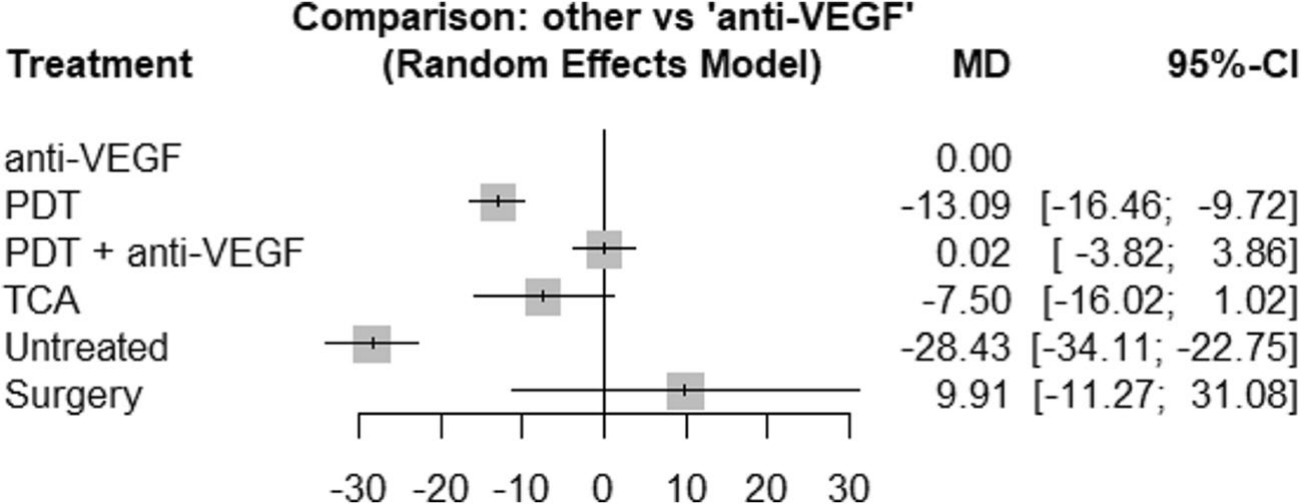
Forrest plot comparing change in BCVA after six months in the anti-VEGF treatment group compared to the other treatment groups. CI, confidence interval; MD, mean difference; PDT, photodynamic treatment; TCA, intravitreal triamcinolone acetonide; VEGF, vascular endothelial growth factors

Concerning the long-term results of BCVA, the evidence network consists of 16 studies, comparing five different treatments as well as no treatment (see Fig. 6). In the anti-VEGF treatment group, the early outcome could be maintained in the long-term analysis with a mean estimated gain of 28.4 letters (95% CI, 22.7–34.1) when compared to untreated patients (*p* < 0.0001). Patients treated with anti-VEGF gained 13.1 letters (95% CI, 9.7–16.5) more than patients treated with PDT (*p* < 0.0001) and 7.5 letters (95% CI, − 1.0–16.0) more than patients treated with TCA, although this was not significant (*p* = 0.084). There was no significant difference between the anti-VEGF group and the combination (PDT and anti VEGF) group (− 0.02; 95% CI, − 3.9–3.8; *p* = 0.991). Also, the gain of 9.91 letters (95% CI, − 11.27–31.08) in the surgical group compared to anti-VEGF treatment stayed not significant (see Fig. 7). We did not observe inconsistency in the network (*p* = 0.328).

**Fig. 8:**
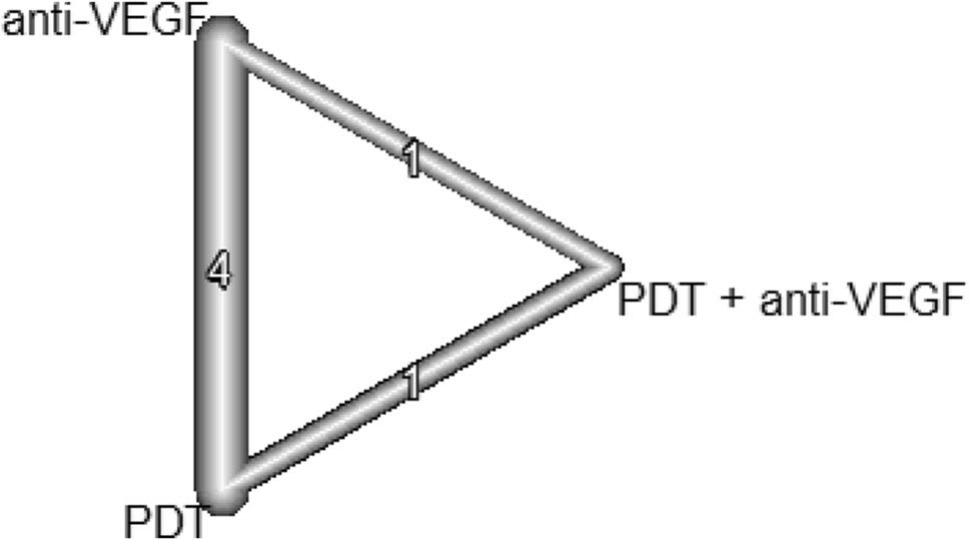
The structure of the network comparing different treatments regarding BCVA in the early phase (< 6 month). The numbers represent the numbers of direct comparisons, while the thickness of the lines is proportional to the inverse standard error of the estimates. PDT, photodynamic treatment; VEGF, vascular endothelial growth factors

**Fig. 9:**
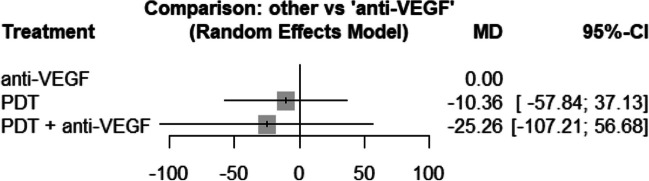
Forrest plot comparing change in central retinal thickness after six months in the anti-VEGF treatment group compared to the other treatment groups. CI, confidence interval; MD, mean difference; PDT, photodynamic treatment; TCA, intravitreal triamcinolone acetonide; VEGF, vascular endothelial growth factors

Central retinal thickness in the later phase was compared using 5 studies with three treatments. Therefore, the network structure shows a triangle shape (see Fig. 8). The network meta-analysis showed no significant difference in the anti-VEGF group compared to the PDT group (10.4 μm; 95% CI, − 37.1–57.8) and no difference to the combination (PDT and anti-VEGF) group (25.3 μm; 95% CI, − 56.7–107.2) (see Fig. 9). Again, this network did not show signs of inconsistency (*p* = 0.447).

Patients treated with 1 + PRN anti-VEGF gained 0.7 letters (95% CI, − 2.3–3.8, *p* = 0.635) compared to the patients treated by 3 + PRN, and their CRT decreased by 3.2 (95% CI, − 15.1–21.4, *p* = 0.734).

### Differences in anti-VEGF drugs

We compared the change in BCVA of different anti-VEGF drugs in the early phase including 8 studies and in the later phase including 13 studies. There was no significant difference in letters gained in patients receiving bevacizumab compared to aflibercept (*p* = 0.222), ranibizumab (*p* = 0.124), and conbercept (*p* = 0.572) in the early phase, the same was seen in the later phase (*p* = 0.250, *p* = 0.265, respectively, *p* = 0.382).

For CRT, we investigated 5 studies for both time points. In the early phase, CRT decreased significantly in patients receiving aflibercept compared to bevacizumab (12.1 μm; 95% CI, 3.0–21.2; *p* = 0.009). There was no significant difference in the change of CRT between bevacizumab, ranibizumab (7.6 μm; 95% CI, − 13.3–28.5), and conbercept (− 5.4 μm; 95% CI, − 41.5–30.8). Moreover, there was also no significant difference observed comparing long-term results of the different anti-VEGF factors.

### Treatment strategies

4 studies compared 1 + PRN and 3 + PRN treatment strategies. Patients treated with 1 + PRN received 1.8 (SD 1.3) injections within 12 months, while patients with 3 + PRN received 3.2 (SD 0.9) injections (*p* < 0.0001).

Also, the number of injections in patients receiving PDT + anti-VEGF versus solely anti-VEGF was compared. Patients receiving combination treatment required 2.2 (SD 1.5) injections, and patients receiving only anti-VEGF treatment required 2.6 (SD 1.3). This difference was not significant (*p* = 0.155).

### Other treatments

Other treatment options for myopic CNV had too few comparators for our quantitative analysis. A summary statement for each option is given in our supplementary table.

## Discussion

This network meta-analysis showed that the intravitreal injection of anti-VEGF using the regimen of 1 + PRN is an effective treatment for myopic CNV, with both short- and long-term beneficial results.

Intravitreal injection of anti-VEGF is considered the gold standard treatment for myopic CNV, which is confirmed in this network meta-analysis. In diabetic macular edema, aflibercept is proposed to lead to a greater improvement in visual acuity compared to other VEGF inhibitors in patients with low baseline BCVA (< 69 letters) [24]. Therefore, we compared the different VEGF inhibitors, i.e., bevacizumab, ranibizumab, aflibercept, and conbercept. However, we found no difference between the VEGF inhibitors. Aflibercept led to a larger decrease in CRT, but this had no impact on visual acuity. Due to small sample sizes, we did not differentiate between low and high baseline BCVA. Future research should investigate this.

We then compared the different treatment strategies for VEGF inhibitors. There was no significant difference in letters gained whether three injections were administered consecutively as loading dose or only one. However, patients treated with 1 + PRN required significantly less injections than patients with 3 + PRN. This outcome might indicate that the 3 + PRN treatment strategy leads to an overtreatment. Future research should investigate in subgroup analysis, whether this is true for different VEGF inhibitors.

Combining anti-VEGF treatment with PDT showed a slightly greater decrease in CRT in the early phase, although the absolute difference of 12 μm may be clinically insignificant. There was a tendency to gain more estimated letters, but this was not significant. In the long-term results (> 6 months), change in BCVA and CRT was the same for anti-VEGF treatment and the combination of PDT and anti-VEGF. There was no difference between these two groups in the number of injections required within 12 months. Considering the absence of randomized controlled trials and the lack of differing results, anti-VEGF monotherapy seems the more reasonable first line treatment.

Intravitreal TCA was inferior to anti-VEGF in terms of letters gained in the short-term analysis, but no statistical difference was seen in long-term analysis. Intravitreal TCA is known to cause an IOP increase in nearly one-third of all patients and has a high prevalence of cataract formation and progression over time. In regard of these known side effects, anti-VEGF appears to be the more favorable choice.

When comparing the previous gold standard PDT for myopic CNV to anti-VEGF, patients with PDT gained significantly less letters over all time periods. This strengthens the use of anti-VEGF over PDT.

In our systematic review, it seems unlikely that other treatment options for myopic CNV show similar visual improvement compared to intravitreal VEGF inhibitors, although patient numbers were too small to prove this in our quantitative network meta-analysis (see supplementary table 2).

The numbers of complications were too small to calculate the risk of complications. In Table 2, we reported complications rates, which were low in general. Surgical interventions had the highest complication rates. Intravitreal steroids, as known, showed an increase of intraocular pressure and cataract progression. In patients with intravitreal VEGF inhibitors, some patients showed corneal erosions and dry eye symptoms after injection. Not all studies reported on these relatively common adverse events, which is the reason why no numbers can be given. The same applies to IOP elevation, as most studies did not measure IOP after injection. There were three (0.001%) reports of retinal detachment after intravitreal injection and one (0.0004%) case of sterile vitritis in the studies reporting on complications.

This network meta-analysis has several limitations. The included studies showed a high degree of heterogeneity of patients’ characteristics, most likely attributable to differences in inclusion and exclusion criteria (see Table 1). Some studies included pretreated patients, while other studies included only treatment-naïve patients. Furthermore, there exists no clear definition of pathologic myopia, and so the studies included slightly different patient populations. Some studies did not report on the definition of myopic CNV used in their study, making a comparison even more difficult. Another very relevant exclusion criteria for intravitreal treatment is the history of vitreous surgery. Again, some studies excluded these patients explicitly, while others included them. As the search was limited to publications in English, we might have missed some studies. However, based on visual inspection of funnel plots and analytical methods, we did not observe signs of publication bias. Further, databases were searched for specific keywords, which did not include all treatment options (for example, laser photocoagulation).

Databases were searched for the following keywords: “myopic choroidal neovascularization”.

Another limitation of this study was the different reporting times of the studies e.g., some studies reported on results after one month, three months, or six months. As our sample size would have been too small to compare the exact time points, we had to pool the different follow-up data under the assumption that the different time points were effectively the same. To make the results more comparable, we gave priority to certain time points, i.e., 3 months, then 6 months and 1 month in the early phase, and 24 months, then 12 months, and as a last option, all follow-up time points after 24 months in the late phase. However, the classification of follow-up dates might bias our results. Further, not all studies used the EDTRS charts for visual acuity testing, and we had to calculate the letter score from other scales. Different OCT devices were used for measuring the central retinal thickness in the studies, making comparison difficult. Additionally, few studies reported CRT as an outcome, which weakens the validity of our results.

Another major limitation of this network meta-analysis is the inclusion of non-randomized trials, which could lead to potential bias within each study. In addition, the inclusion of RCTs and observational studies could result in study designs and data collection which are not comparable.

## Conclusion

This network meta-analysis shows that intravitreal VEGF inhibitors are the most effective treatment of myopic CNV with few adverse events and a preferred treatment regimen of 1 + PRN.

### Supplementary Information

Below is the link to the electronic supplementary material.

Supplementary file1 (PDF 248 KB)
