# Supplementary material for: Treatment of myopic choroidal neovascularization: a network meta-analysis and review
Source: Graefes Arch Clin Exp Ophthalmol. 2023 Nov 11;262(6):1693–722. doi: 10.1007/s00417-023-06271-2 (PMC11106160; doi:10.1007/s00417-023-06271-2)
Supplement: Supplementary file 1 — Supplementary file1 (PDF 248 KB) [file 417_2023_6271_MOESM1_ESM.pdf]

Supplementary Table 1.

Section/Topic	Item # *	Checklist Item†	Reported on Page #
**TITLE**			
Title	1	Identify the report as a systematic review *incorporating a network meta-analysis (or related form of meta analysis).*	Page 1
**ABSTRACT**			
Structured summary	2	Provide a structured summary including, as applicable:<br>Background: main objectives<br>Methods: data sources; study eligibility criteria, participants, and interventions; study appraisal; and *synthesis methods, such as network meta-analysis.*<br>Results: number of studies and participants identified; summary estimates with corresponding confidence/credible intervals; *treatment rankings may also be discussed. Authors may choose to summarize pairwise comparisons against a chosen treatment included in their analyses for brevity.*<br><br>Discussion/Conclusions: limitations; conclusions and implications of findings.<br>Other: primary source of funding; systematic review registration number with registry name.	Page 3, Abstract
**INTRODUCTION**			
Rationale	3	Describe the rationale for the review in the context of what is already known, *including mention of why a network meta-analysis has been conducted.*	Page 4, Introduction
Objectives	4	Provide an explicit statement of questions being addressed, with reference to participants, interventions, comparisons, outcomes, and study design (PICOS).	Page 4, Introduction
**METHODS**			
Protocol and Registration	5	Indicate whether a review protocol exists and if and where it can be accessed (e.g., Web address); registration and, if available, provide registration information, including registration number.	Page 5, Methods (literature search)
Eligibility criteria	6	Specify study characteristics (e.g., PICOS, length of follow-up) and report characteristics (e.g., years considered, language, publication status) used as criteria for eligibility, giving rationale. *Clearly describe eligible treatments included in the treatment network, and note whether any have been clustered or merged into the same node (with justification).*	Page 5, Methods (literature search, study eligibility criteria and study selection)
Information sources	7	Describe all information sources (e.g., databases with dates of coverage, contact with study authors to identify additional studies) in the search and date last searched.	Page 5, Methods (Literature search)
Search	8	Present full electronic search strategy for at least one database, including any limits used, such that it could be repeated.	Page 5, Methods (Literature search)
Study selection	9	State the process for selecting studies (i.e., screening, eligibility, included in systematic review, and, if applicable, included in the meta-analysis).	Page 5-7 Methods (study eligibility criteria, Study selection, Data extraction, Data analysis)<br>Page 21 Figure 1

Data collection process	10	Describe method of data extraction from reports (e.g., piloted forms, independently, in duplicate) and any processes for obtaining and confirming data from investigators.	Page 5-6 Methods (Data extraction)
Data items	11	List and define all variables for which data were sought (e.g., PICOS, funding sources) and any assumptions and simplifications made.	Page 5-6 Methods (Data extraction)
Geometry of the network	S1	Describe methods used to explore the geometry of the treatment network under study and potential biases related to it. This should include how the evidence base has been graphically summarized for presentation, and what characteristics were compiled and used to describe the evidence base to readers.	Page 6 and 7 Data analysis; Page 22, 24, 26, 28 (Figure 2,4,6,8)
Risk of bias within individual studies	12	Describe methods used for assessing risk of bias of individual studies (including specification of individual studies whether this was done at the study or outcome level), and how this information is to be used in any data synthesis.	Discussion
Summary measures	13	State the principal summary measures (e.g., risk ratio, difference in means). *Also describe the use of additional summary measures assessed, such as treatment rankings and surface under the cumulative ranking curve (SUCRA) values, as well as modified approaches used to present summary findings from meta-analyses.*	Page 6 and 7 Methods (Data analysis)
Planned methods of analysis	14	Describe the methods of handling data and combining results of studies for each network meta-analysis. This should include, but not be limited to:<br><br>*Handling of multigroup trials;*<br>*Selection of variance structure;*<br>*Selection of prior distributions in Bayesian analyses; and*<br>*Assessment of model fit.*	Page 6 and 7 Methods (Data analysis)
Assessment of inconsistency	S2	Describe the statistical methods used to evaluate the agreement of direct and indirect evidence in the treatment network(s) studied. Describe efforts taken to address its presence when found.	Page 6and 7 Methods (Data analysis)
Risk of bias across studies	15	Specify any assessment of risk of bias that may affect the cumulative evidence (e.g., publication bias, selective reporting within studies).	Page 6and 7 Methods (Data analysis) Page 13 and 14 Discussion
Additional analysis	16	Describe methods of additional analyses if done, indicating which were prespecified. This may include, but not be limited to, the following:<br><br>Sensitivity or subgroup analyses;<br>Meta-regression analyses;<br>*Alternative formulations of the treatment network; and*<br>*Use of alternative prior distributions for Bayesian analyses (if applicable).*	Page 6 and 7 Methods (Data analysis)
**RESULTS‡**			
Study selection	17	Give numbers of studies screened, assessed for eligibility, and included in the review, with reasons for exclusions at each stage, ideally with a flow diagram.	Page 8 (Results) and Page 21(Figure 1)
Presentation of network structure	S3	Provide a network graph of the included studies to enable visualization of the geometry of the treatment network.	Page 22, 24, 26, 28 (Figure 2,4,6,8)
Summary of network structure	S4	Provide a brief overview of characteristics of the treatment network. This may include commentary on the abundance of	Page 8-11 (Results)

		trials and randomized patients for the different interventions and pairwise comparisons in the network, gaps of evidence in the treatment network, and potential biases reflected by the network structure.	
Study characteristics	18	For each study, present characteristics for which data were extracted (e.g., study size, PICOS, follow-up period) and provide the citations.	Page 5-6 Methods (Data extraction), Page 30-56 Table 1 and 2
Risk of bias within studies	19	Present data on risk of bias of each study and, if available, any outcome level assessment.	Page 13,14 Results
Results of individual studies	20	For all outcomes considered (benefits or harms), present, for each study: 1) simple summary data for each intervention group, and 2) effect estimates and confidence intervals. *Modified approaches may be needed to deal with information from larger networks.*	Page 30-56 Table 1 and 2
Synthesis of results	21	Present results of each meta-analysis done, including confidence/credible intervals. *In larger networks, authors may focus on comparisons versus a particular comparator (e.g., placebo or standard care), with full findings presented in an appendix. League tables and forest plots may be considered to summarize pairwise comparisons.* If additional summary measures were explored (such as treatment rankings), these should also be presented.	Page 8-11 Results
Exploration for inconsistency	S5	Describe results from investigations of inconsistency. This may include such information as measures of model fit to compare consistency and inconsistency models, *P* values from statistical tests, or summary of inconsistency estimates from different parts of the treatment network.	Page 6-7 Methods Data analysis, Results
Risk of bias across studies	22	Present results of any assessment of risk of bias across studies for the evidence base being studied.	
Results of additional analyses	23	Give results of additional analyses, if done (e.g., sensitivity or subgroup analyses, meta-regression analyses, *alternative network geometries studied, alternative choice of prior distributions for Bayesian analyses,* and so forth).	Page 8-10 Results
**DISCUSSION**			
Summary of evidence	24	Summarize the main findings, including the strength of evidence for each main outcome; consider their relevance to key groups (e.g., health care providers, researchers, and policymakers).	Page 12-14 Discussion
Limitations	25	Discuss limitations at study and outcome level (e.g., risk of bias), and at review level (e.g., incomplete retrieval of identified research, reporting bias). *Comment on the validity of the assumptions, such as transitivity and consistency. Comment on any concerns regarding network geometry (e.g., avoidance of certain comparisons).*	Page 13,14 Discussion
Conclusions	26	Provide a general interpretation of the results in the context of other evidence, and implications for future research.	Page 12-14 Discussion
**FUNDING**			
Funding	27	Describe sources of funding for the systematic review and other support (e.g., supply of data); role of funders for the systematic review. This should also include information regarding whether funding has been received from manufacturers of treatments in the network and/or whether some of the authors are content experts with professional conflicts of interest that could affect use of treatments in the network.	Page 1

Supplementary Table 2.

**Treatment options with too few comperators**	**Summary Statement**
Laser photocoagulation	Parodi et al [22] examined the use of laser photocoagulation for myopic choroidal neovascularization (CNV). In the group receiving laser treatment, there was a insignificant decrease in BCVA from 62.5 letters to 57 letters after two years. In contrast, the group receiving anti-VEGF treatment showed an increase in BCVA from 54.5 letters to 64 letters. Laser photocoagulation may have some short-term effectiveness, but observational studies indicate that the formation of atrophic laser scars could potentially have negative consequences[25].
Surgical intervention	Two studies [19, 20] reported on surgical interventions. In the study by Glacet-Bernard [19], BCVA improved in the translocation group (n=32) after three months from 35 to 55 letters, which was a greater gain compared to the PDT treatment group (n=34). The study by Hamelin[20] observed an increase of 48.5 letters in the surgical removal group (n=18) with a mean follow-up of 14 months and a gain of 31 letters in the translocation group (n=14) with a mean follow-up of 11 months. Both studies reported on high treatment costs, serious complications related to the surgery and a high percentage of CNV recurrence [20].
Oral bisphosphonates	A study by Miki et al [21] investigated the effect of oral bisphosphonates on myopic CNV. Bisphosphonates are known to have an antiangiogenic impact by suppressing VEGF expression [26]. Miki et al [21] reported a significant increase in BCVA in the bisphosphonate group (21 eyes, +14 letters, p=0.032) compared to the untreated group. However, eyes treated with anti VEGF (37 eyes, +19.5 letters) showed better results. Since common side effects of long-term bisphosphonates use include hypocalcemia, bone pain, and osteonecrosis of the jaw, bisphosphonate treatment does not seem a reasonable alternative.

Radiotherapy	Kobayashi et al [7] compared radiotherapy in 20 eyes to 19 untreated eyes. BCVA slightly increased from 37.1 letters at baseline to 38.9 letters after 3 months and then decreased to 36.1 letters after two years. In the untreated group, BCVA decreased after two years.
Sub-Tenon TCA	Wakabayashi et al [23] investigated the use of sub-Tenon TCA and compared it to bevacizumab. In the 20 eyes receiving sub-Tenon TCA BCVA worsened by 1.5 letters, while the 34 eyes receiving bevacizumab improved by 9 letters ($p<0.001$).
